# Exome first approach to reduce diagnostic costs and time – retrospective analysis of 111 individuals with rare neurodevelopmental disorders

**DOI:** 10.1038/s41431-021-00981-z

**Published:** 2021-10-25

**Authors:** Julia Klau, Rami Abou Jamra, Maximilian Radtke, Henry Oppermann, Johannes R. Lemke, Skadi Beblo, Bernt Popp

**Affiliations:** 1 Institute of Human Genetics, University of Leipzig Medical Center, Leipzig, Germany; 2 Center for Rare Diseases, University of Leipzig Medical Center, Leipzig, Germany

**Keywords:** Epilepsy, Neurodevelopmental disorders, Genetic testing, Genetics research, Paediatrics

## Abstract

This single-center study aims to determine the time, diagnostic procedure, and cost saving potential of early exome sequencing in a cohort of 111 individuals with genetically confirmed neurodevelopmental disorders. We retrospectively collected data regarding diagnostic time points and procedures from the individuals’ medical histories and developed criteria for classifying diagnostic procedures in terms of requirement, followed by a cost allocation. All genetic variants were re-evaluated according to ACMG recommendations and considering the individuals’ phenotype. Individuals who developed first symptoms of their underlying genetic disorder when Next Generation Sequencing (NGS) diagnostics were already available received a diagnosis significantly faster than individuals with first symptoms before this cutoff. The largest amount of potentially dispensable diagnostics was found in genetic, metabolic, and cranial magnetic resonance imaging examinations. Out of 407 performed genetic examinations, 296 (72.7%) were classified as potentially dispensable. The same applied to 36 (27.9%) of 129 cranial magnetic resonance imaging and 111 (31.8%) of 349 metabolic examinations. Dispensable genetic examinations accounted 302,947.07€ (90.2%) of the total 335,837.49€ in potentially savable costs in this cohort. The remaining 32,890.42€ (9.8%) are related to non-required metabolic and cranial magnetic resonance imaging diagnostics. On average, the total potentially savable costs in our study amount to €3,025.56 per individual. Cost savings by first tier exome sequencing lie primarily in genetic, metabolic, and cMRI testing in this German cohort, underscoring the utility of performing exome sequencing at the beginning of the diagnostic pathway and the potential for saving diagnostic costs and time.

## Introduction

Neurodevelopmental disorders (NDD) and epilepsy are frequent causes for medical presentations/diagnostics [1, 2]. NDD affect ~3% of children and ~1/150 develop epilepsy in the first ten life years [3, 4]. Genetically they are heterogeneous including hundreds of disorders [5, 6].

Establishing a diagnosis involves coordinated interplay between pediatricians, neurologists, geneticists, and other physicians in centers for rare diseases. This process is time consuming, requires many diagnostic procedures and burdens affected families [7–10]. The associated costs and risks are considerable, underscoring the scope of this “diagnostic odyssey” and the load on the health care system [11]. The impact of a conclusive genetic diagnosis for individuals/families has been investigated in several studies and demonstrates the importance of developing effective diagnostic guidelines [7, 12–14].

Genetic diagnostics have traditionally been initiated at a relatively late stage in the diagnostic pathway and were often performed in a stepwise manner [15, 16]. Since its introduction, high-throughput sequencing has proven to be an effective, rapid but cost-intensive method in genetic diagnostics [17] and has evolved from a research method to a routine diagnostic tool [18]. Next Generation Sequencing (NGS) outperforms traditional genetic diagnostics, as it can achieve a diagnostic yield between 30 and 47% in NDD and epilepsy cohorts combined with speed-up through a single test [16, 19–22]. Thus, the question remains [23] whether first-line exome sequencing (ES) could save costs and time, reduce the risks of obsolete extensive or even invasive diagnostic procedures, and allow families to make earlier reproductive decisions.

To address these questions, we designed a retrospective single-center study focusing on diagnostic procedures and time durations in 111 individuals with NDD and/or epilepsy with NGS based diagnosis. We wanted to determine the extent to which NGS diagnostics influenced the diagnostics duration and to assess the number of diagnostic interventions that might have not been required retrospectively if ES had been implemented earlier in this cohort.

## Materials and methods

### Study design and patient selection

**Fig. 1:**
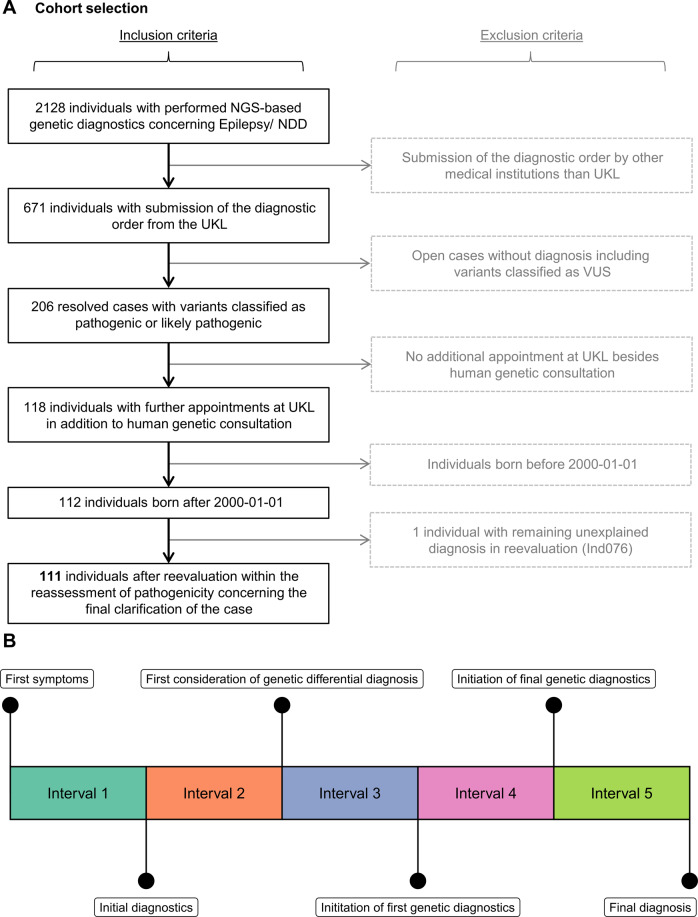
Flowchart of participant recruitment and timeline. **A** Workflow used to select individuals for this study. Further information on the excluded individual (Ind076) is provided in supplementary files. **B** Schematic timeline, time points and intervals (compare Supplementary methods for color coding) of the diagnostic trajectory researched from the patient’s medical history.

We retrospectively selected 111 individuals with pediatric-onset NDD and/or epilepsy from a diagnostic cohort of 2128 cases based on a series of filtering and quality control steps (see details in Fig. 1A). All individuals had received a molecular diagnosis using NGS methods at the Institute of Human Genetics at Leipzig University Medical Center (UKL), a tertiary care centre in Germany, between 2017-04-04 and 2020-08-03. Our filtering criteria were intended to ensure that physician letters were available for all individuals to collect data on diagnostic procedures as well as time points of interest. Therefore, we excluded individuals who received no clinical assessments at UKL and those born before the year 2000. We excluded cases where only variants of uncertain significance (VUS) had been reported in the initial diagnostic report. This resulted in a list of 112 individuals for which we collected clinical data. We excluded one individual after variant reevaluation because the reported variant does not sufficiently explain the observed severity (Ind076; for further details see Supplementary File S3).

### Data collection and analysis

The UKLs patient information systems were used to curate genetic/clinical information from the individuals’ medical history. This involved all procedures performed from birth to the final molecular report. We classified each procedure by its requirement. Only the final NGS-based investigations leading to diagnosis and their validation was classified as required for genetic analyses. We recorded diagnostic time points (t1–t6) on the structured diagnostic pathway (Fig. 1B). Associated diagnostic costs were determined using a retrospective bottom-up approach by inferring the total health care costs based on individual procedures. Data were compiled in Excel (Microsoft Corporation, Redmond, Washington, USA) and analyzed in RStudio (Version 1.4). See Supplementary methods for details.

### Genetic analyses and variants reevaluation

All individuals were diagnosed by NGS-methods such as gene panel, single ES or trio ES and variants were re-evaluated to current standards (see details in Supplementary methods).

## Results

### Cohort demographics

**Fig. 2:**
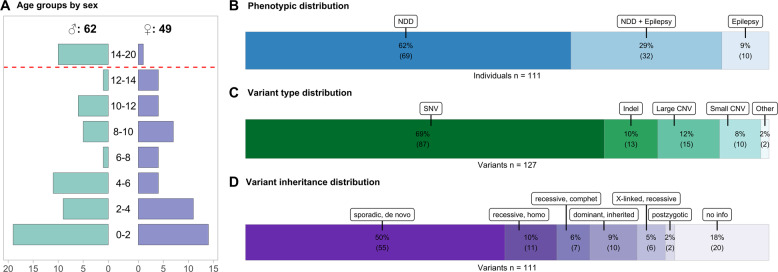
Individual and variant features. **A** Divergent plot representing the age distribution of individuals. The age determination refers to the date of final establishment of the human genetic diagnosis. The *y*-axis represents the age classes in years. The *x*-axis shows the number of associated individuals. **B** Stacked bar chart indicating the distribution of the phenotypes (epilepsy, NDD and the combination of both) in the 111 included individuals presented in percentages. **C** Stacked bar chart showing the distribution of all variant types found in the individuals including additional findings seen in no or low association with NDD or epilepsy presented in percentages (*n* = 127). SNV = Single-nucleotide variant, Indel = Insertion/Deletion Polymorphism, Large CNV = Copy number variation whose alteration affects multiple genes, Small CNV = Copy number variation whose alteration affects a single gene and involves, for example, several exons (**D**). Stacked bar chart showing the inheritance patterns of those variants assigned to the respective phenotype of epilepsy or NDD (*n* = 111). homo = homozygous, comphet = compound heterozygous.

Of the 111 individuals included, 49 (44.1%) were female and 62 (55.8%) were male (ratio: 1:1.26). Due to the inclusion criteria described earlier (Fig. 1A), all individuals enrolled were under 20 years of age at the time of diagnosis. Details of the sex-specific age distribution are provided in Fig. 2A. Initial clinical diagnosis was NDD in 69 (62.1%) of individuals, epilepsy in 10 (9%), and a combined occurrence of both in 32 (28.8%) (Fig. 2B). In ten individuals with isolated epilepsy the seizure onsets and genetic diagnoses occurred in a very young age, or no medical records were available for the time after genetic diagnosis. We examined further medical letters for possible later onsets of NDD, but no evidence for it was found. Thus, it cannot be excluded that NDD could still develop in these individuals later in life. The onset of the first symptoms associated with the underlying genetic disease developed at a median age of 6.2 months (range: 0–156.6; standard deviation (SD): 20.5) in the individuals. Out of 111 individuals 22 individuals (19.8%) were already symptomatic regarding the molecular diagnosis on their day of birth and 50 individuals (45%) showed symptoms in their first year of life; another 39 individuals (35.1%) became symptomatic in their second year of life or later. A compilation of anonymized individual data is provided in Supplementary File S2.

### Variant characteristics

In 111 individuals, a total of 127 variants was reported through NGS-based diagnostics. The majority of 87 (68.5%) variants were single-nucleotide variants (SNV), with the remainder composed of 15 (11.8%) copy number variants affecting multiple genes (Large CNV), 13 (10.2%) insertion/deletion variants (indel), ten (7.9%) copy number variation whose alteration affects a single gene and involves several exons (Small CNV), and two complex events (1.6%) summarized as “other” (Fig. 2C). These comprise an unbalanced translocation identified through coverage-based CNV analysis and subsequently confirmed by karyotyping and FISH and a deletion-insertion event in *MECP2* (Variant ID: CNV017 and SNV008; Supplementary File S3). Of these 127 variants, 111 variant combinations represented as causative for the phenotypes of NDD/epilepsy in the individuals, whereas the other genetic alterations were related to other concerns or were incidental findings. The origin of most NDD/epilepsy-related variants was recorded as sporadic and de novo in 55 cases (49.5%). The inheritance of eleven variants (9.9%) was recessive and homozygous, ten (9%) were dominant and inherited, another seven (6.3%) variants combinations represented as recessive compound heterozygous variants, six (5.4%) were X-linked recessive and two (1.8%) were postzygotic. For 20 variants (18%) no segregation was performed in the parents (Fig. 2D). A detailed compilation of all identified variants is provided in Supplementary File S3.

### Time periods in the diagnostic pathway

The total diagnostic time (first symptoms to final diagnosis) was split into subintervals (Interval 1–5; Fig. 1B) to allow granular analysis of the diagnostic trajectory. The median duration between onset of symptoms associated with the underlying genetic disease and the final genetics report establishing the diagnosis was 34.1 months (range: 0.6–210.5; SD: 57.4). A median of 64.5 days (range: 8–395; SD: 60.8) passed between the initiation of NGS-based diagnostics leading to molecular diagnosis and the report of the molecular diagnosis.

**Fig. 3:**
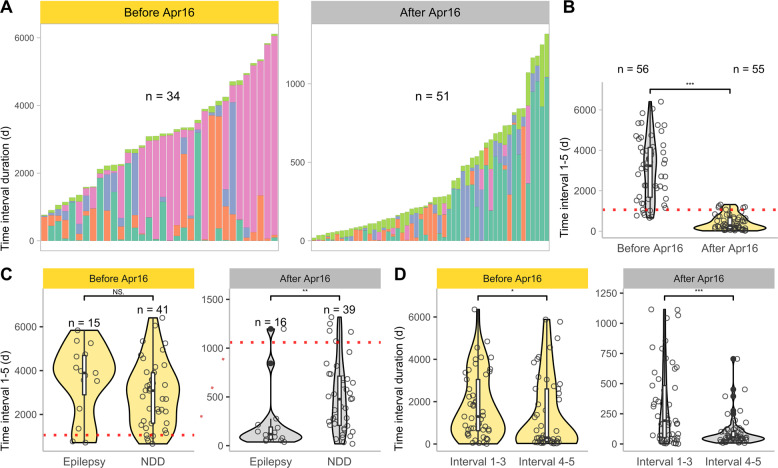
Impact established NGS-based diagnostics on diagnostic time intervals. **A** Stacked bar plot showing the length of each diagnostic subinterval (Interval 1, 2, 3, 4, and 5) per individual, grouped by time of first symptom onset. Cohort is subdivided by availability of NGS-based diagnostics after April 1, 2016. Only individuals for which the duration of all diagnostic intervals could be calculated were included for this figure (*n* = 84). Colors as in Fig. 1B. **B** Violin- and scatter-plot of the total diagnostic time interval (Interval 1–5, first symptoms to final diagnosis) grouped by first symptom onset. (*p* < 0.001, Wilcox-Test). **C** Violin- and scatter-plot assembling the total diagnostic time interval (Interval 1–5) grouped by phenotype according to the time of onset of first symptoms. Individuals with a combination of both phenotypes were assigned to the phenotype that occurred first. (After Apr16: *p* ~ 0.002, Wilcox-Test). **D** Violin- and scatter-plot assembling Interval 1–3 (first symptoms to initiation of first genetic diagnostics) and Interval 4–5 (initiation of first genetic diagnostics to final diagnosis) grouped by onset symptoms. (After Apr16: *p* < 0.001, Wilcox-Test).

We compared the length of subintervals in individuals with first symptoms before (*n* = 56) and after (*n* = 55) the availability of NGS-based diagnostics at UKL in April 2016 (Fig. 3A). While Interval 4 (initiation of first genetic diagnostics to initiation of final genetic diagnostics) dominates in the total diagnostic duration for individuals with symptom onset before the establishment of NGS, Interval 1 (first symptoms to initial diagnostics) emerges prominently for individuals with first symptoms after April 2016. The duration of the total diagnostic trajectory (Interval 1–5) significantly (*p* < 0.001, Wilcox-Test) differed between individuals with first symptoms before and after 2016-04-01 (Fig. 3B); the median interval length was 106.3 months (range: 21.7–201.5; SD: 50.2) for individuals with first symptoms before establishment of NGS, whereas it was shorter for individuals with first symptoms thereafter, with a median of 9.2 months (range: 0.6–43.3; SD: 12.1). This is expected because recruitment of individuals ended in August 2020. Thus, the total diagnostic time interval length for individuals with first symptoms after the NGS introduction is limited to a maximum of 4 years.

Dividing these subgroups based on initial phenotype (NDD or epilepsy first), no significant differences emerged for individuals with initial symptoms before April 2016. For individuals with first symptoms after 2016, those in the “epilepsy first” group received their molecular diagnosis significantly faster (*p* ~ 0.002, Wilcox-Test; 3.9 months; range: 0.13–36.53; SD: 10.6) than individuals in the “NDD first” group (15.7 months; range: 0.62–43.3; SD: 12) (Fig. 3C).

To compare the proportion of the total diagnostic interval pertaining to pediatrics with that of clinical genetics, we attributed Interval 1–3 (pediatrics) and 4–5 (genetics) to them. While only a relatively weak significant difference (*p* ~ 0.03, Wilcox-Test) was found for individuals with first symptoms before April 2016, a highly significant difference (*p* < 0.001, Wilcox-Test) was found for individuals with first symptoms after 2016. For these individuals, the time allocated to pediatrics (193 days; range: 1–1115; SD: 326.4) was significantly longer than that attributed to genetics (54 days; range: 8–705; SD: 123.2) (Fig. 3D, Supplementary Fig. S3).

The duration of the total diagnostic pathway did not differ between individuals whose initial diagnosis took place at UKL and individuals whose initial diagnosis was performed at another medical institution (Supplementary Fig. S4).

### Diagnostic procedures and costs

**Fig. 4:**
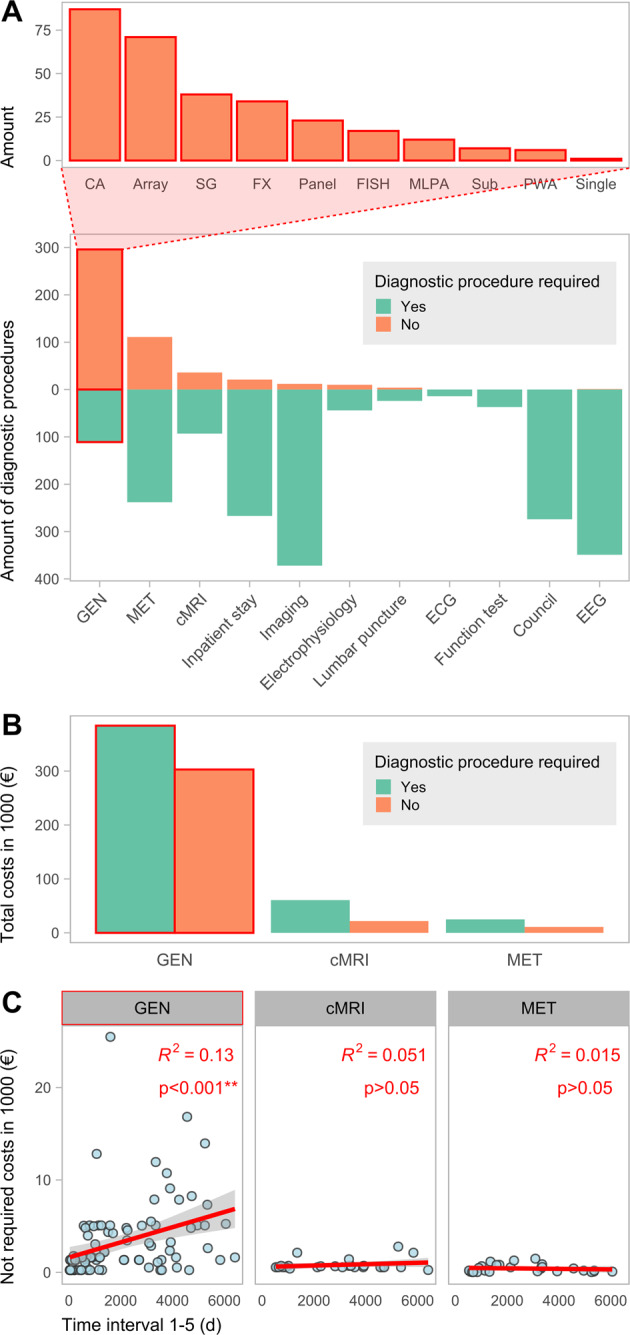
Required and non-required diagnostic costs. **A** Divergent plot illustrating the amount of required and not required diagnostics performed on the individuals. GEN = human genetic diagnostics, MET = metabolic laboratory testing, cMRI = cranial magnetic resonance imaging, Imaging = other medical imaging than cMRI, ECG = electrocardiogram, EEG = electroencephalogram. The bar chart above categorizes the non-required genetic diagnostics. CA = Chromosome analysis, SG = Single Gene analysis, FISH = Fluorescence in situ hybridization, MLPA = Multiplex ligation-dependent probe amplification, Sub = Analysis of Subtelomeres, PWA = Prader-Willi syndrome and Angelman syndrome diagnostics, Single = Single Exome Diagnostics. **B** Bar chart showing the respective costs grouped by top three diagnostic categories (with the highest amount of potential non-required diagnostics) and requirement. Compare Supplementary Fig. S7 for a cost calculation that excludes external costs. **C** Scatter-plot showing the correlation of the respective unnecessary diagnostic costs with the length of the diagnostic odyssey (Interval 1–5), grouped by diagnostic category. The values for *R*^2^ and the *p* values are given in the plot.

Before receiving a genetically confirmed diagnosis, the individuals in our cohort underwent various diagnostic procedures (Fig. 4A, Supplementary File S2). All individuals received at least one genetic examination, 101 individuals (91%) had at least one laboratory test other than metabolic testing and 86 individuals (77.5%) received at least one cMRI. Other medical imaging was performed at least once in 78 (70.3%) individuals, medical consults in 75 (67.6%), EEG in 74 (66.7%), metabolic diagnostics in 67 (60.4%), lumbar puncture in 26 (23.4%), electrophysiology in 26 (23.4%), functional tests in 14 (12.6%), ECG in 11 (9.9%). Overall, 95 individuals (85.6%) were in inpatient care at UKL at least once (Supplementary Fig. S5).

The diagnostic categories with the highest amounts of potentially non-required procedures are genetic diagnostics, metabolic diagnostics, and cMRI (Fig. 4A). Of 407 genetic examinations performed overall, 296 (72.7%) were categorized as potentially dispensable if an exome-wide analysis would have been performed initially instead. Among those 407 examinations, 314 (77.1%) were performed at UKL and 93 (22.8%) at other hospitals. Based on our study design, all genetic examinations performed outside the UKL were considered as non-essential. The different types of dispensable genetic diagnostics are mainly constituted by chromosomal analyses, arrays, single gene testing, fragile X syndrome diagnostics and small custom gene panels (Fig. 4A, top part). In total 129 cMRI examinations were performed in this cohort, of which 36 (27.9%) were categorized as not required. With one exception, all of those were performed under general anesthesia. Contrast agent was used in 18 (50%) of dispensable imaging procedures. Of 349 metabolic examinations, 111 (31.8%) were categorized as dispensable. Of these 129 cMRI examinations, 94 (72.9%) were performed at UKL and 35 (27.1%) at other hospitals. We categorized 26 cMRI performed at UKL (27.7%) as potentially not required, whereas of 35 cMRI performed elsewhere (28.5%) were considered as dispensable. Only a minority of procedures in the categories of laboratory tests other than metabolic tests, medical imaging except cMRI, electrophysiology, and EEG were classified as non-essential. All consults, ECG, functional tests, EEG examinations were classified as essential. We classified 21 (7.3%) of a total of 288 inpatient stays performed as potentially dispensable. Among these, non-required hospitalizations had a median of two inpatient overnight stays (range: 1–7; SD: 1.9) and were significantly shorter (*p* ~ 0.016, Wilcox-Test) than hospitalizations classified as indispensable, with a median length of four nights (range: 0–731; SD: 46.7) (Supplementary Fig. S6).

We calculated the costs for the three diagnostic categories with the largest number of non-required diagnostic procedures: genetic diagnostics, metabolic diagnostics, and cMRI. In our cohort, a total of 687,168.02€ was spent on genetic diagnostics. Thereof, 302,947.07€ (44.1%) are associated with dispensable examinations. Of the 82,589.20€ spent on cMRI, 21,903.37€ (26.5%) were considered not required if the final genetic diagnosis would have been known and considered. From 35,980.43€ issued for metabolic examinations, a portion of 10,987.05€ (30.5%) was classified as not required (Fig. 4B). Thus, genetic examinations show the highest cost savings potential with 302,947.07€ (90.2%) out of 335,837.49€.

On average, the total potentially savable cost amounts to 3025.56€ per individual in our study. This corresponds to an average of 2729.25€ for genetic diagnostics, 197.33€ for cMRI examinations and 98.98€ for metabolic testing regarding potential cost savings.

The amount of summed dispensable cost per individual does not correlate with the length of the diagnostic trajectory (Interval 1–5) for metabolic diagnostics and cMRI. In genetic diagnostics, a moderate positive correlation (*p* < 0.001, Pearson correlation coefficient = 0.367, *R*^2^ = 0.13) is observed between the amount of the summed costs of genetic testing per individual and the length of time needed to obtain a diagnosis (Fig. 4C).

## Discussion

This retrospective cohort analysis emphasizes the importance of implementing ES as a first line test in the diagnostic pathway for individuals with NDD and/or epilepsy. NGS-based testing ended the hitherto inconclusive diagnostic pathway in all 111 individuals in our study. Therefore, the length of the diagnostic odyssey is significantly shorter in individuals with first symptoms onset after the availability of NGS than in individuals developing first symptoms before this cut-off time. It must be considered that our study included only individuals who received a final molecular diagnosis, and we can thus not assess individuals who had inconclusive NGS-based diagnostics. However, our design is a representative snapshot of the currently achievable diagnostic yield (~31–53%) using ES as a first-tier test [24] in NDD/epilepsy.

Even before the broad availability of NGS in the clinical setting, genetic testing in this cohort was often initiated early by the treating pediatricians. However, the diagnostic odyssey was prolonged by the unavailability of genetic diagnostics covering the considerable heterogeneity observed in NDD and epilepsy (SysID database [25] accessed on 2021-04-24 states 1454 genes associated with NDD whereof 663 were associated with epilepsy). This caused a stepwise evaluation using karyotyping, microarrays, and clinically suspected diagnoses. After the establishment of NGS, this phase receded, while the time interval before initiation of genetic diagnostics became critical for a fast diagnosis. Direct comparison of Interval 1–3 (pediatricians) and 4–5 (medical geneticists) before and after clinical availability of NGS demonstrates a remarkable reduction on the genetics side. This shows the accelerating impact of NGS-based methods on diagnostic time intervals. Thus, further potential for shortening the diagnostics lies in the faster referral of individuals with NDD/epilepsy to genetics testing. We recommend early human genetics consults in the pediatric diagnostic process, which could also be implemented using telemedical methods. A close and coordinated cooperation between pediatricians and human geneticists is essential to achieve a fast diagnosis. These measures are generalizable and will likely improve time to diagnosis in any health care system.

Our data imply that individuals who first presented at other or smaller hospitals had no disadvantages in terms of time to molecular diagnosis. This may be due to good cooperation between institutions or the well-known role of our Center for Rare Diseases in the local area.

Individuals with epilepsy received a quicker diagnosis than those with NDD after establishment of clinical NGS. Thus, the decrease in diagnostic duration is more apparent in individuals with epilepsy. This might be explained by the assumption that pediatricians may be more sensitive to the possible genetic background of epilepsy. The potentially more acute clinical presentation of epileptic seizures could also have led to a more rapid initiation of genetic diagnostics. Furthermore, the focus on epilepsy research in genetics at UKL may have contributed to this. It would be desirable if this could be increasingly established for NDD entities without epilepsy.

Stark et al. [26]. and Tan et al [27] examined the cost-effectiveness of NGS and also considered potential cost savings by omitting other traditional diagnostic procedures. These studies did however not consider whether these diagnostic interventions might nevertheless be indispensable in individual cases. Soden et al. [28] analyzed potentially dispensable medical costs through rapid ES in NDDs, but included diagnostics that would have been performed even if the genetic diagnosis was known in their cost savings model. Therefore, our careful reassessment of each individual case, represents the first study to examine the extent of diagnostics that could retrospectively be replaced by early implementation of ES based on accepted criteria for diagnostics of NDD and epilepsy in a tertiary Center for Rare Diseases.

The greatest potential for cost savings concerns prior genetic diagnostics in our study. Of the genetic tests performed, 72.7% were classified as potentially dispensable representing 90.2% of total savable costs. A reversal from classical genetic diagnostics to an exome first approach [29] would thus have resulted in reduced diagnostic costs in our cohort. Particularly because ES is also increasingly suitable for the detection of CNVs (in our cohort 26 CNVs, including small and complex alternations like translocations, were identified through NGS), which provides an alternative to chromosomal microarrays [22, 30–32]. After inconclusive first tier ES, genetic examinations could be extended accordingly to entities possibly not covered, such as translocations, repeat disorders (fragile X syndrome) or mitochondrial disorders.

The proportion of dispensable metabolic (31.8%) and cMRI examinations (27.9%) is relevant, because genetic testing can compete with, or even surpass them in terms of diagnostic yield [31, 33]. ES prior to performing these diagnostics should therefore be considered. Unlike other studies [34] we did not classify all metabolic diagnostics as dispensable, because they are essential confirmatory diagnostics and used for therapy decisions. Furthermore, metabolic testing currently provides diagnostic results in critical situations faster than genetic testing. Faster genetic results in the future could therefore replace additional metabolic examinations.

Our data suggest that the potential for diagnostic savings through ES lies primarily in genetic diagnostics. This is an effect of the granulated and stepwise approach performed historically. Except for metabolic and cMRI examinations, there was little to no potential for further savings.

A comparison of the savable costs in our cohort with the results of other studies is limited, because of differences in study design and cohort selection. Tan et al. [27] and Stark et al. [26] prospectively designed different diagnostic pathways in the course of ES in an undiagnosed cohort and compared their estimated costs. Based on this, cost savings per additional diagnosis (inflated and currency converted values: 6237.14€ and 1561.58€) were determined. Soden et al. provide the mean costs of prior negative testing in non-acute individuals (inflated and currency converted value: 17,785.18€). Chung et al. [35], Monroe et al. [34], and Vrijenhoek et al. [20] calculated the cost savings per individual resulting from avoidable medical examinations due to early ES implementation (inflated and currency converted values: 110.63€; 3277.66€, and 5090.39€). In some of these publications the costs of potentially unnecessary diagnostics even exceeded costs for ES. In our data, the cost of ES amounts to 3461.45€ while the average cost savings by avoidable diagnostics is 3025.56€ per individual. Thus, the average cost of potentially dispensable diagnostics almost reaches the cost level of an ES examination. The avoidable costs in this study are lower than in some previously publications. This may be an effect of our cautious evaluation of potentially dispensable diagnostics and our focus on solely direct diagnostic costs. Furthermore, previous studies report higher costs for individual parameters of metabolic diagnostics as well as for cMRI which could be explained by different pricing policies of health care systems in other countries.

Hospitalizations that we classified as potentially avoidable were shorter than indispensable inpatient stays. This can be explained by the solely diagnostic purpose of these not required hospitalizations, whereas required hospitalizations were mostly associated with complex therapies, emergency admissions, or a poor general condition of the individuals. While only 7.3% of inpatient stays (with a median of only two nights) were classified as dispensable, their potential omission could have been a major relief for affected families in individual cases [36].

Costs alone should not determine the course of action in rare disease care. Early implementation of ES could reduce diagnostic costs and time, but also prevent exhausting or even risky procedures. In our study, more than a quarter of cMRI examinations were judged to be potentially dispensable and most were performed using anesthesia. Putting a child at risk [37] for imaging with sedation and contrast agent could be influenced by the outcome of prioritized genetic testing since imaging results rarely lead to diagnosis in individuals with NDD [38]. A quick molecular diagnosis can also affect therapy and medical interventions [13, 22, 27], have an impact on family planning [12, 16], and contribute to the psychological well-being of the parents [14].

Moreover, our study demonstrates the importance of reanalyzing and reevaluating exome data (compare Table 1 and Supplementary results) [12, 39]. The reassessment of the *SMAD4* variant initially reported as incidental finding in in Ind012 is of decisive clinical importance for the affected family who now do not have to partake invasive colonoscopies anymore. In addition, our reevaluation revealed one individual in which the previously reported genetic diagnosis could not sufficiently explain the observed phenotype because the associated phenotype was too mild. The formal downgrading of five variants is expected and an effect of continuous development and stricter interpretation of the ACMG criteria for variant classification. We considered the five cases as resolved by the plausibility of these “hot VUS”. Future analyses might facilitate a definitive assessment as reevaluation in light of new guidelines and expanding genetic databases may yield new results [40].

**Table 1:** Information of the five individuals with “hot VUS” variants after reevaluation.

Variant ID	Individual ID	Gene	Variant	Variant classification	Classification rules	Zygosity	Origin
SNV_002	Ind002	*TUBB2B*	c.611 A > T, p.(Asn204Ile)	VUS	PM2_Supporting, PP2_Supporting, PP3_Supporting, PP4_Supporting	Heterozygous	Maternal
SNV_022	Ind019	*NPRL3*	c.434 T > C, p.(Leu145Pro)	VUS	PS2_Moderate, PM2_Supporting, PP3_Supporting	Heterozygous	de novo (confirmed)
SNV_027	Ind025	*DYNC1H1*	c.8945 G > A, p.(Arg2982His)	VUS	PS2_Moderate, PM2_Supporting, PP2_Supporting, PP3_Supporting	Heterozygous	de novo (confirmed)
SNV_067	Ind069	*SCN1A*	c.4581 + 18 A > G, p.?	VUS	PS2_Moderate, PM2_Supporting, PP3_Supporting	Heterozygous	de novo (confirmed)
SNV_073	Ind077	*NBEA*	c.4662 + 1 G > C, p.Thr1519_Val1554del	VUS	PVS1_Moderate, PS2_Moderate, PM2_Supporting	Heterozygous	de novo (confirmed)

Limitations of this study include the relatively small cohort, the lack of control group by focusing on solved cases only and the retrospective approach. Our study design included data on examinations from hospitals other than UKL to increase the number of available data for these important categories. We used the GOÄ billing system for consistent cost calculation to minimize potential bias through this approach. Also, only a part of dispensable costs was determined, so the extent of these may potentially be higher. Overall, some of the study design choices were imposed by the billing system and decentralized medical system in Germany.

Further studies on this topic should involve larger cohorts in a prospective setting. Collaboration among multiple rare disease centers and more complete collection of individuals’ medical data across the boundaries of single medical institutions would benefit for this research aim. However, individuals with rare NDD and/or epilepsy entities will surely benefit from continued development and research into rapid and effective diagnostic pathways. Therefore, close and informed collaboration between different medical specialties, such as pediatrics and human genetics, is essential. Both early consideration of a genetic differential diagnosis and quick performance of ES can contribute to reduce diagnostic time, costs and exhausting medical procedures and enable a sooner reproductive choice in the families.

## Supplementary information

Supplementary File S1

Supplementary File S2

Supplementary File S3

## Data Availability

All data generated or analyzed during this study are included in this published paper and its supplementary information files.
