# Supplementary material for: Exome first approach to reduce diagnostic costs and time – retrospective analysis of 111 individuals with rare neurodevelopmental disorders
Source: Eur J Hum Genet. 2021 Oct 25;30(1):117–25. doi: 10.1038/s41431-021-00981-z (PMC8738730; doi:10.1038/s41431-021-00981-z)
Supplement: Supplementary file 1 — Supplementary File S1 [file 41431_2021_981_MOESM1_ESM.docx]

**SUPPLEMENTARY NOTES:**

**Exome first approach to reduce diagnostic costs and time – retrospective analysis of 111 individuals with rare neurodevelopmental disorders**

Julia Klau^1^, Rami Abou Jamra^1^, Maximilian Radtke^1^, Henry Oppermann^1^, Johannes R. Lemke^1,2^, Skadi Beblo^2,*^, Bernt Popp^1,*^

^1^ Institute of Human Genetics, University of Leipzig Medical Center, Leipzig, Germany

^2^ Center for Rare Diseases, University of Leipzig Medical Center, Leipzig, Germany

* These authors contributed equally to this work

**SUPPLEMENTARY METHODS**

**Clinical data collection and classification**

The internal patient information systems of the UKL were used to curate genetic and clinical information from the individuals' medical history. We collected data on all diagnostic measures i.e. genetic analyses, cranial magnetic resonance imaging (cMRI), metabolic diagnostics (defined as analyses of parameters in a blood, urine or liquor sample, such as organic acids, amino acid profiles, acylcarnitines, etc.), other laboratory diagnostics, lumbar punctures, other medical imaging than cMRI (including sonographic, endoscopic, X-ray and computer tomography examinations), electrocardiograms (ECG), electroencephalograms (EEG), electrophysiology, function tests (including spirometry, polysomnography and cardiorespirogram examinations) and conciliar examinations. Metabolic parameters examined in cerebrospinal fluid samples were assigned to metabolic diagnostics. The lumbar puncture itself and the associated laboratory tests concerning, for example, inflammatory parameters are included in the lumbar puncture category. For details on the specific types of the respective diagnostic procedures, see Supplemental File S2 (sheet “costs”). We also included hospitalisations and counted the amount of inpatient overnight stays. For laboratory tests other than metabolic diagnostics, we only recorded whether they were performed at least once due to the amount of single data points with neglectable overall costs. We concentrated on the diagnostics performed at UKL excluding additional examinations potentially performed at other medical institutions. This approach is in-line with previous studies^1^ and served to ensure a uniform approach because, especially in the case of individuals with a longer medical history at other hospitals, our access to physician letters was restricted. However, we included data on cMRI and genetic examinations performed in other medical institutions, because these information were consistently transmitted to the UKL. Our data collection involved all procedures performed from birth to the report of the final molecular diagnosis. We classified each diagnostic entry according to its requirement considering the individual medical history and the exact circumstances of each specific situation based on assessment by pediatricians experienced with rare diseases. Many diagnostic interventions were regarded as indispensable, especially for critically ill individuals or when warning signs like a reduced general condition, developmental regression, and new onset seizures were present. Our classification system was divided into three parts and contains the categories "not required", "required" and "ambiguous". All genetic investigations were classified as dispensable, except for the final NGS-based investigations leading to the diagnosis and their subsequent validation by Sanger, MLPA, and/ or q/RT-PCR. The curated classification criteria for all procedures are provided in Supplemental File S2 (sheet “criteria”).

**Diagnostic time data collection**

We recorded diagnostic time points for each individual based on the structured diagnostic pathway as depicted schematically in Figure 1B. Data for the following time points were collected: occurrence of first symptoms associated with the genetic phenotype (t1), initial diagnostics related to the underlying genetic disease (t2), first consideration of a genetic differential diagnosis (t3), initiation of first genetic diagnostics (t4), enrollment of the NGS-based examination that lead to the final diagnosis (t5) and the corresponding diagnosis report date (t6).

Color coding of the intervals used in the main Figures is: Interval 1 (turquoise) includes the time period between the onset of the first symptoms and the initial diagnostics. Interval 2 (orange): initial diagnostics to first consideration of genetic differential diagnosis. Interval 3 (blue): first consideration of genetic differential diagnosis to initiation of first genetic diagnostics. Interval 4 (pink): initiation of first genetic diagnostics to initiation of final genetic diagnostics. Interval 5 (green): initiation of final genetic diagnostics to final diagnosis.

**Cost calculation**

We determined associated diagnostic costs using a retrospective bottom-up approach by inferring the total cost to the health care system from determining the cost of individual procedures. We focused on direct costs incurred solely due to diagnostic procedures. Other direct medical costs as well as indirect costs were not considered. The cost calculation is based on the “Gebührenordnung für Ärzte” (GOÄ), which determines the billing of medical costs in the outpatient sector in Germany. Even though many diagnostic measures performed on the individuals in our cohort occurred in a hospital setting, we nevertheless calculated them on the basis of the GOÄ. In Germany, costs incurred during hospitalizations are billed as [flat-rate payment](https://www.linguee.de/englisch-deutsch/uebersetzung/flat-rate+payment.html)s via Diagnosis Related Groups (DRGs), which does not allow conclusions to be drawn about particular diagnostic costs.^3^ Therefore, we also did not include the costs of hospitalization in our cost calculations to avoid duplications. We focused the cost analysis on the three categories with the largest amounts of non-required procedures, which were genetic diagnostics, cMRI, and metabolic diagnostic costs. Our estimated costs for gene panel sequencing, trio ES and single ES were fixed to 3461.45€ per examination based on the GOÄ cost system in September 2020 including accounting of the nucleic acids isolation from the blood sample, sequencing and scientific evaluation and report. This cost level is consistent with estimated costs of NGS-based diagnostics in other studies. Schwarze et al.^2^ report a range of costs for ES between 555$ and 5,169$ (inflated value in €: 507,79€ - 4729,30€); Vrijenhoek et al.^3^ state 3,600€ (inflated value: 3,693.27€) for ES consistent with our costs. We inflated the costs for comparative purposes using a web inflation tool (https://www.inflationtool.com) and converted them subsequently to Euro using corresponding daily exchange rates from Bloomberg L.P. on 2021-05-06 (for conversion and inflation calculation of costs see Supplementary Table S1). A specific diagnostic costs summary is available in Supplementary File S2 (sheet “costs”).

**Genetic diagnostics**

By study design all 111 individuals received their molecular diagnosis by NGS-based genetic diagnostics. Four individuals (3.6%) received an in-house custom-design panel targeting genes associated with epilepsy (“Epi-panel”, 122 genes). TruSight One v1 Panel (Illumina, Inc., San Diego, CA) was performed in 68 individuals (61.3%) which includes 4,811 genes associated with human disease. Whole exome sequencing (WES) targeting all coding genes was performed in 38 individuals (34.2%) using either a BGI Exome capture 59M kit (ten individuals; BGI, Shenzhen, China), SureSelect Human All Exon V6 (four individuals; Agilent Technologies, Santa Clara, CA, USA) or a TWIST Human Core Exome Kit (25 individuals; TWIST Bioscience, San Francisco, CA, USA) target design. For all included individuals, the genomic regions targeted by the respective enrichment design had an average coverage of ≥ 100 reads and ≥ 95% were covered by ≥ 10 reads.

**Genetic Analyses and Variants Reevaluation**

To increase sample size, we included individuals diagnosed by gene panel examinations in our cohort because we assumed the identified variants were definitively diagnostic and would have been detected by exome sequencing as well.^4^ Only those individuals with variants classified as pathogenic or likely pathogenic and considered to fully cause the phenotype were selected for this study. We performed a reevaluation of all detected variants according to the updated guidelines provided by the American College of Medical Genetics and Genomics (ACMG)^5^ to standardize and update all variant pathogenicity assessments. For consistent variant nomenclature and standardized evaluation, web resources like ClinVar (https://www.ncbi.nlm.nih.gov/clinvar/), Varsome (https://varsome.com/), Decipher (https://www.deciphergenomics.org/), Mutalyzer (<https://mutalyzer.nl>), gnomAD (https://gnomad.broadinstitute.org/), ClinGen Pathogenicity Calculator (https://cnvcalc.clinicalgenome.org/cnvcalc/) and AutoPVS1 (<http://autopvs1.genetics.bgi.com/>), were used. We ensured that all variants reported here were submitted to ClinVar (see Supplementary File S3 for ClinVar IDs).

**Statistical analysis and Data Plotting**

We analysed and graphically processed the data compiled in Excel (Microsoft Corporation, Redmond, Washington, USA) using R language (Version 4.0.5) from within RStudio (Version 1.4). Because of its infrequent application, the diagnostic entries that were marked in the "ambiguous" category were assigned to the "required" category for our statistical analysis following a conservative evaluation approach. In order to make comparisons of diagnostic times intervals we subdivided the cohort. This division was based on the onset time of first symptoms concerning the genetic phenotype before and after the introduction of NGS-based diagnostics. This cut-off was set to April 1, 2016, the date since when NGS based diagnostics where broadly applied at our Institute of Human Genetics in Leipzig. We used two sided Wilcoxon signed-rank test as implemented in R to calculate p-values for groups differences.

**SUPPLEMENTARY RESULTS**

**Variants Reevaluation**

Reevaluation resulted in downgrading of one variant previously reported as pathogenic and exclusion of one individual from subsequent analyses (Ind076; Supplemental File S3) leaving 111 individuals for our final cohort. The variant is a microdeletion (CNV_013) identified in Ind076 which does not adequately explain the highly disabled individual's phenotype in its severity. This individual deceased on the fourth day of life after uncontrollable seizures and severe asphyxia. The microdeletion identified, which includes SIN3A among others, is associated with milder disease cases.6 Because of the challenging accessibility of this case due to the early death of the individual, we decided to exclude this case from our calculations, as we cannot fully clarify it. After reassessment of all variants formerly classified as (likely) pathogenic, 106 out of 111 phenotype-related variants remained in those assessment categories (Supplemental File S3). Five variants had to be downgraded to VUS (Table 1). This can be explained inter alia by the change in recommendations regarding the PM2 criterion. A ClinGen7 recommendation to modify the strength of the PM2 criterion “Absent from controls, or at extremely low frequency if recessive, in Exome Sequencing Project, 1000Genomes Project, or Exome Aggregation Consortium”5 resulted in a downgrade from moderate to supportive. The PS2 criterion was betimes judged to be too strong in some but can only be assessed with strength moderate due to unspecific phenotype in most NDD entities and lack of data on the occurrence of this variant in other individuals. For further verification, RT-PCR would have been necessary for Ind069 to confirm the splice effect of the de novo variant c.4581+18A>G in SCN1A. Because these five VUS were estimated to be very plausible with the phenotype, we nevertheless considered these cases resolved (“hot VUS”) and retained these individuals in the cohort. Further investigation and more information on other individuals with the same variants will prospectively help to improve the assessment of these cases in the future. The re-evaluation of a SMAD4 duplication initially reported as incidental finding in Ind012 (Supplemental File S3, Supplemental Figure S1 and S2) showed that this was a retrotransposition event rather than a true genomic duplication as initially assumed.

**SUPPLEMENTARY FIGURES**


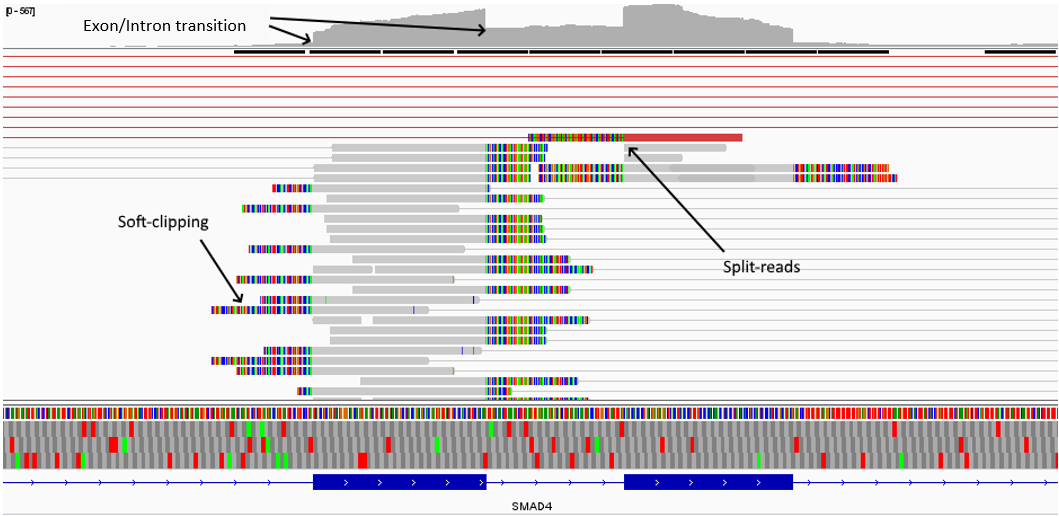
**Figure S1 Sequencing data of the *SMAD4* gene in Ind012 with retrotransposition-
 typical reads in exons 5 and 6**

Sequencing data from individual Ind012 concerning questionable alterations in *SMAD4* gene. The variant previously reported as a duplication of exons 2,3 and 5-12 in the *SMAD4* gene is a retrotransposition and not a duplication of genomic DNA as originally thought. The sequence fragments ("reads") were visually inspected in the IGV browser. Here we observed that only the coding regions (including exon 4), but not the introns, show more of these reads. These unusual reads start in one exon and end in another ("split reads") or show a particular pattern in the mapping to the reference sequence with abrupt end of the sequence at the exon-intron transition ("soft-clipping"). This indicates the absence of introns and the presence of a spliced transcript at the DNA level. The additional reads still cause the bioinformatics algorithm to indicate a duplication. Taken together, this suggests the presence of a retrotransposon, which is characterized by the reverse transcription of processed mRNA with subsequent insertion into the genome and thus involves only the exons. These types of retrotransposons insert randomly into the genome, where they are very unlikely to be transcribed due to the lack of regulatory sequences. Furthermore, it is extremely unlikely to insert in the intact SMAD4 gene and disrupt its function here. In most cases, such retrotransposons are inherited, functionless and present in the non-coding region of the genome. In rare cases, it can insert into areas of other genes and thus interfere with their translation or transcription. For methodological reasons, the insertion point can not be determined by the method used. Alterations in *SMAD4* gene can cause the juvenile polyposis syndrome.^8^ The identification of the *SMAD4* variant as a retrotransposition removes the need for preventive examinations regarding polyps in the gastrointestinal system of the individual and its family. A correction report was submitted to the affected family based on the re-assessment in our study.

**Figure S2 Position of the inconspicuous MLPA probe in intron 4 of the *SMAD4* gene**


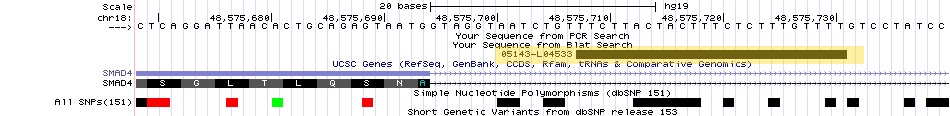


Moreover, the confirmation reported in our previous finding by a second method (MLPA Kit P158, company MRCHolland) and the observed absence of a duplication of exon 4 can be explained by the presence of a retrotransposition. Indeed, the probe for exon 4 in this kit ("05143-L04533", sequence: TTCTTACTACTT-TCTCTTTGTTTT) is located 24 base pairs after the exon-intron transition and is therefore not indicated as duplicated.

**Figure S3 Proportions of Interval 1-3 and Interval 4-5 in the total diagnostic interval (Interval 1-5)**

**
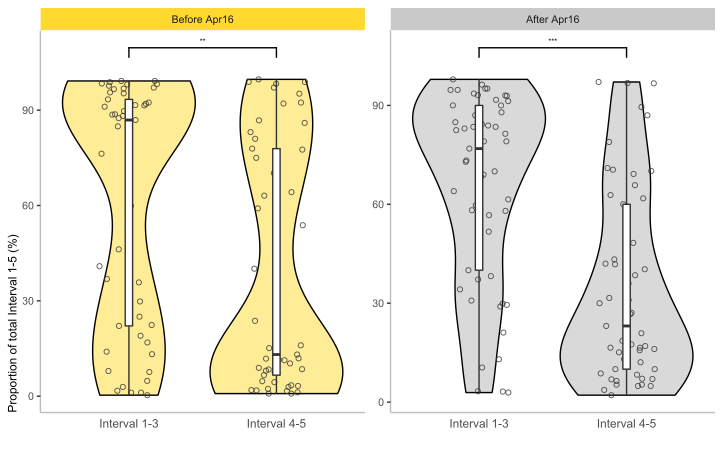
**

Violin- and scatter-plot showing the percentages of interval 1-3 and interval 4-5 within the total diagnostic interval grouped by the onset of first symptoms. The grouping was based on the establishment NGS-based human genetic diagnostics and labelled as before April 1, 2016 (Before Apr16) and after April 1, 2016 (After Apr16). Wilcoxon test was used for significant testing with significant results.

**Figure S4 Total diagnostic time interval grouped by the location of the initial diagnosis
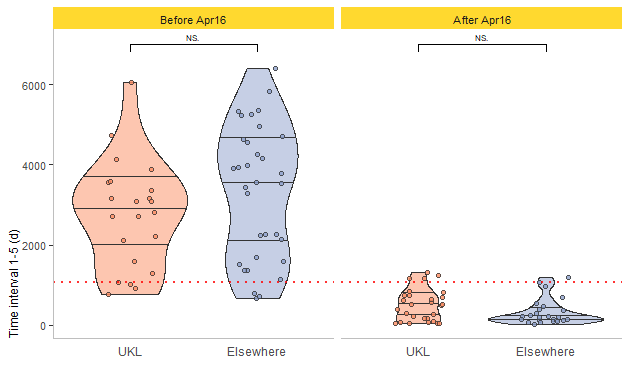
**

Violin- and scatter-plot assembling the total diagnostic time interval (Interval 1-5, first symptoms to final human genetic diagnosis) grouped according to the time of onset of first symptoms. The grouping was based on the establishment NGS-based human genetic diagnostics and labelled as before April 1, 2016 (Before Apr16) and after April 1, 2016 (After Apr16). The red dashed line marks the median of the total group. Wilcoxon test was used for significant testing with a non-significant result.


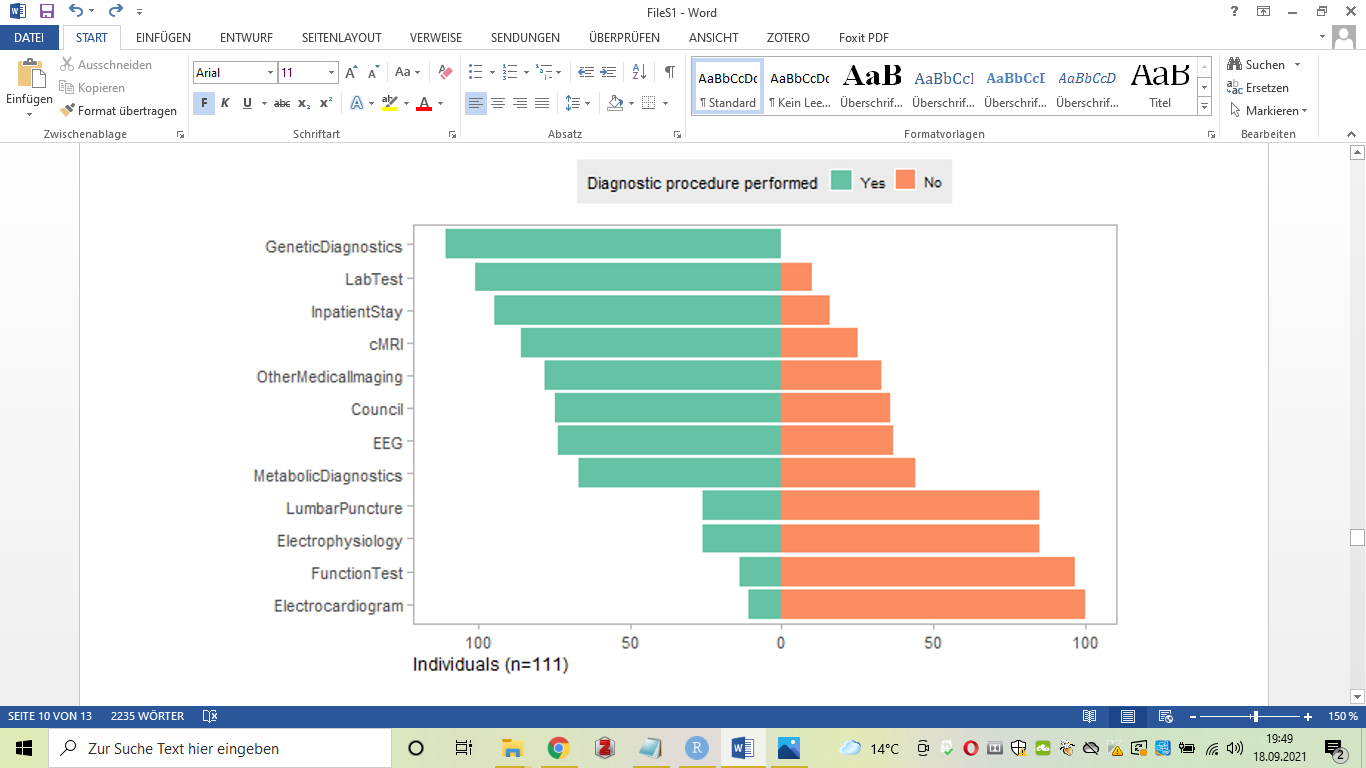
**Figure S5 Amount of performed diagnostic procedures**

Divergent bar plot showing the number of diagnostic measures performed and not performed. The left side in green indicates the proportion of the 111 individuals for whom the respective diagnostic procedure was performed at least once. The right side in red shows the remaining proportion of the cohort for whom the respective diagnostic procedure was never performed.

**Figure S6 Comparison of length of required and non-required inpatient stays**


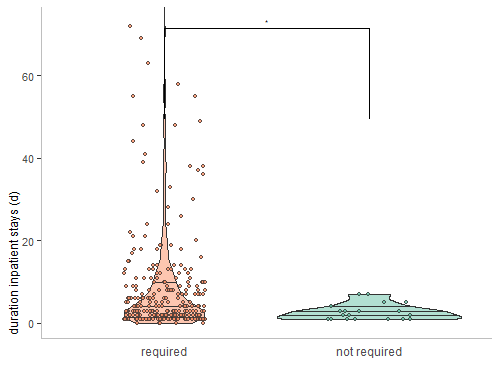


Violin- and scatter-plot assembling the length of hospital stays in days grouped according to our classification into required and potentially waivable inpatient stays. Wilcoxon test was used for significant testing with a weak significant result.

**
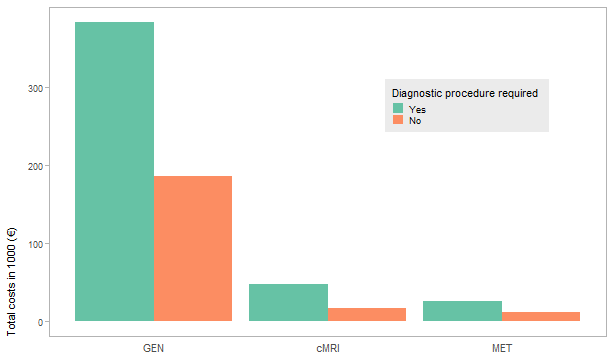
Figure S7 Required and non-required diagnostic costs excluding external costs**

Bar chart showing the respective costs grouped by top three diagnostic categories (with the highest amount of potential non-required diagnostics) and requirement. Only the diagnostic costs incurred at the UKL were taken into account for this figure. Diagnostic costs for human genetic and cMRI examinations at other hospitals were excluded. A total of 570,128.52€ was spent on genetic diagnostics. Thereof, 185,907.57€ (32.6%) are associated with dispensable examinations. Of the 64,146.23€ spent on cMRI, 16,633.95€ (25.9%) were considered not required if the final genetic diagnosis would have been known and considered. From 35,980.43€ issued for metabolic examinations, a portion of 10,987.05€ (30.5%) was classified as not required. This results in a total of 213,528.57€ in potential savings, with a substantial proportion of the costs for genetic diagnostics which amount to 185,907.57€ (87.06%). This results in a possible cost savings of 1,923.68€ per individual. This corresponds to an average of 1,674.84€ for genetic diagnostics, 149.86€ for cMRI examinations and 98.98€ for metabolic testing regarding potential cost savings. Certainly, the savable costs are lower not including external diagnostic data, but the costs of human genetic testing continue to be the major component.

**ABBREVIATIONS**

ACMG: American College of Medical Genetics; cMRI: cranial magnetic resonance imaging; CNV: copy number variant; DRGs: Diagnosis Related Groups; ECG: electrocardiogram; EEG: electroencephalogram; ES: exome sequencing; GOÄ: Gebührenordnung für Ärzte; MLPA: multiplex ligation-dependent probe amplification; NDD: neurodevelopmental disorder; NGS: Next Generation Sequencing; RT-PCR: reverse transcription polymerase chain reaction; SD: standard deviation; SNV: single-nucleotide variant; UKL: Leipzig University Medical Center; VUS: variants of uncertain significance

**SUPPLEMENTARY FILES**

**File S1:** Supplementary notes with supplementary figures and tables.

**File S2:** Comprehensive tabular data describing relevant clinical cohort information.

**File S3:** Information on all variants identified in this cohort.

**SUPPLEMENTARY TABLES**

**Table S1 Cost savings from other studies and their inflated values for 2021 in €**

| **Study** | **Publication submission year** | **Cost type** | **Original costs reported** | **Inflated costs for 2021** | **Inflated costs converted to €** |
| --- | --- | --- | --- | --- | --- |
| Tan et al.^9^ | 2017 | *cost savings per additional diagnosis* | US$6,838.00 | US$7,488.00 | €6,237.14 |
| Soden et al.^1^ | 2014 | *average cost of prior negative tests in non-acute patients* | US$19,100.00 | US$21,352.00 | €17,785.18 |
| Chung et al.^10^ | 2020 | *avoided healthcare costs* | HKD$1,005.53 | HKD$1034.26 | €110.63 |
| Monroe et al.^11^ | 2015 | *average cost savings for genetic and metabolic investigations in diagnosed patients* | US$3,547.00 | US$3,935.00 | €3277.66 |
| Stark et al.^12^ | 2016 | *cost savings per additional diagnosis* | US$1,702.00 | US$1,874.76 | €1561.58 |
| Vrijenhoek et al.^3^ | 2017 | *avoided costs of traditional genotyping technologies, other diagnostic interventions, and all other laboratory investigations* | €4,896.00 | €5,090.39 | €5,090.39 |

**REFERENCES**

1. Soden SE., Saunders CJ., Willig LK., Farrow EG., Smith LD., Petrikin JE., et al. Effectiveness of exome and genome sequencing guided by acuity of illness for diagnosis of neurodevelopmental disorders. *Sci Transl Med* 2014;**6**(265):265ra168-265ra168. Doi: 10.1126/scitranslmed.3010076.

2. Schwarze K., Buchanan J., Taylor JC., Wordsworth S. Are whole-exome and whole-genome sequencing approaches cost-effective? A systematic review of the literature. *Genet Med* 2018;**20**(10):1122–30. Doi: 10.1038/gim.2017.247.

3. Vrijenhoek T., Middelburg EM., Monroe GR., van Gassen KLI., Geenen JW., Hövels AM., et al. Whole-exome sequencing in intellectual disability; cost before and after a diagnosis. *Eur J Hum Genet* 2018;**26**(11):1566–71. Doi: 10.1038/s41431-018-0203-6.

4. Dillon OJ., Lunke S., Stark Z., Yeung A., Thorne N., Gaff C., et al. Exome sequencing has higher diagnostic yield compared to simulated disease-specific panels in children with suspected monogenic disorders. *Eur J Hum Genet* 2018;**26**(5):644–51. Doi: 10.1038/s41431-018-0099-1.

5. Richards S., Aziz N., Bale S., Bick D., Das S., Gastier-Foster J., et al. Standards and guidelines for the interpretation of sequence variants: a joint consensus recommendation of the American College of Medical Genetics and Genomics and the Association for Molecular Pathology. *Genet Med* 2015;**17**(5):405–23. Doi: 10.1038/gim.2015.30.

6. Witteveen JS., Willemsen MH., Dombroski TCD., van Bakel NHM., Nillesen WM., van Hulten JA., et al. Haploinsufficiency of MeCP2-interacting transcriptional co-repressor SIN3A causes mild intellectual disability by affecting the development of cortical integrity. *Nat Genet* 2016;**48**(8):877–87. Doi: 10.1038/ng.3619.

7. Patel RY., Shah N., Jackson AR., Ghosh R., Pawliczek P., Paithankar S., et al. ClinGen Pathogenicity Calculator: a configurable system for assessing pathogenicity of genetic variants. *Genome Med* 2017;**9**(1):3. Doi: 10.1186/s13073-016-0391-z.

8. Hussain T., Church JM. Juvenile polyposis syndrome. *Clin Case Rep* 2020;**8**(1):92–5. Doi: 10.1002/ccr3.2616.

9. Tan TY., Dillon OJ., Stark Z., Schofield D., Alam K., Shrestha R., et al. Diagnostic Impact and Cost-effectiveness of Whole-Exome Sequencing for Ambulant Children With Suspected Monogenic Conditions. *JAMA Pediatr* 2017;**171**(9):855. Doi: 10.1001/jamapediatrics.2017.1755.

10. Chung CCY., Leung GKC., Mak CCY., Fung JLF., Lee M., Pei SLC., et al. Rapid whole-exome sequencing facilitates precision medicine in paediatric rare disease patients and reduces healthcare costs. *Lancet Reg Health - West Pac* 2020;**1**:100001. Doi: 10.1016/j.lanwpc.2020.100001.

11. Monroe GR., Frederix GW., Savelberg SMC., de Vries TI., Duran KJ., van der Smagt JJ., et al. Effectiveness of whole-exome sequencing and costs of the traditional diagnostic trajectory in children with intellectual disability. *Genet Med* 2016;**18**(9):949–56. Doi: 10.1038/gim.2015.200.

12. Stark Z., Tan TY., Chong B., Brett GR., Yap P., Walsh M., et al. A prospective evaluation of whole-exome sequencing as a first-tier molecular test in infants with suspected monogenic disorders. *Genet Med* 2016;**18**(11):1090–6. Doi: 10.1038/gim.2016.1.
